# Effect of adding a positive memories’ module in a trauma-focused cognitive-behavioural treatment for female survivors of intimate partner violence: trial protocol

**DOI:** 10.1186/s13063-022-06540-1

**Published:** 2022-07-23

**Authors:** M. Crespo, A. Miguel-Alvaro, C. Hornillos, S. Sánchez-Ferrer, A. A. Antón

**Affiliations:** Facultad de Psicología, Universidad Complutense de Madrid, Campus de Somosaguas, s/n, 28223 Madrid, Spain

**Keywords:** Intimate partner violence, Posttraumatic stress, Positive memories, Cognitive-behaviour therapy, Trauma-focus, Randomized controlled trial

## Abstract

**Background:**

Trauma-focused cognitive-behavioural treatments have been proven to be effective for reducing symptoms in female survivors of intimate partner violence (IPV), although they still present some difficulties (e.g. significant drop-out rates, low adherence). Based on existing evidence about the difficulty of accessing memories of positive experiences among these women, we considered integrating positive memory evocation in trauma-focused treatments. The present study aims to test the effect of adding a positive memory module to trauma-focused CBT for female survivors of IPV.

**Methods:**

The study is a single-blind, randomized controlled trial (RCT) comparing two trauma-focused CBT (with and without a positive memory module) for female survivors of IPV and a wait-list condition (superiority trial), including pretreatment and posttreatment measures, and follow-ups at 3, 6 and 12 months. Assessors of treatment outcome will be blinded to the trial arm. We aim to recruit 135 participants who will be randomized to one of the experimental conditions. The primary outcome is PTSD symptom severity. Secondary outcome measures include IPV, attitudes towards IPV, posttraumatic cognitions, centrality of trauma, self-concept, positive and negative affect, depression, anxiety, emotional dysregulation or health-related quality of life, as well as satisfaction with treatment. Moreover, adherence to and satisfaction with treatment will be considered.

**Discussion:**

This study first analyses the effect of including positive memory evocation into a trauma-focused treatment for female survivors of IPV. This strategy aims to improve the effect of the treatments and enhance the healing of the trauma by developing a more integrated and emotionally modulated autobiographical narrative that contributes to the recovery and well-being of the victims.

**Trial registration:**

ISRCTN73702156. Registered on 10 March 2022.

## Background

Various cognitive-behavioural treatments focused on trauma have been shown to be effective with a large number of trauma survivors [1, 2]; however, many clinical trials have intentionally excluded women who suffer intimate partner violence (IPV) (e.g. groups of Foa and Resick) for reasons such as alleging that they are treatments only for previous traumas rather than ongoing trauma [3]. Moreover, this lack of interest is related to a series of factors that affect the way in which these treatments can be applied to women who survive IPV and that hinder their accessibility to such treatments. Notably, the concurrence of a series of demands (e.g. protecting children, obtaining financial support, legal processes) that lead to the lack of time and money to follow a treatment or the fear that the aggressor will use against her the fact or following a psychological treatment (e.g. claiming that she is not able to take care of the children or use it as an excuse for the continuation of violence). In short, trauma-focused therapies for victims of IPV are neither desired nor accessible to all survivors. In addition, such therapies do not focus on many of the domains affected by long-term interpersonal trauma, such as IPV.

Until recently, the treatment of traumatic sequelae in women who are victims of continuous intimate partner violence has received little attention. In recent years, a series of reviews have been published focused on psychological interventions among survivors of IPV [3–7]. An analysis of these reviews allows us to identify three psychological intervention programs specifically focused on trauma for women survivors of IPV with contrasted evidence of their effectiveness for their application in a generalized manner (i.e. not focused only on groups of women with specific socioeconomic characteristics, sociocultural characteristics or comorbidities): (1) the Cognitive Trauma Therapy for Battered Women (CTT-BW), which was developed by the Kubany team [8–10]; (2) the Helping to Overcome PTSD through Empowerment (HOPE), which was developed by Johnson and Zlotnick [11] and Johnson et al. [12]; and (3) the Cognitive-Behavioural Treatment (CBT) for battered women with posttraumatic symptom [13, 14].

These three programs involve the application of brief (between 1 and 2 months of total duration) and multiple components interventions that offer a structured description of the treatments and that provide results on their effectiveness, thus highlighting the effect of the program on the reduction of posttraumatic and depressive symptoms over time. However, the analysis of these programs leads us to conclude, as did Warshaw et al. [3] or more recently Hameed et al. [7], that more research is needed to analyse the effectiveness of trauma-focused treatments for women survivors of IPV, especially if they are still being abused or if they are at risk of reabuse. It is important to remember that the treatments designed to reduce PTSD or trauma-related depression were originally created for unique traumatic events or traumatic experiences that occurred in the past and are unlikely to occur in the future. For many women, however, abuse or fear of future abuse is ongoing, regardless of their relationship with the abuser. In these circumstances, some of the components of the treatment may be particularly difficult to tolerate and may require modification. For example, “reliving” abuse through some form of exposure can potentially increase a woman’s discomfort rather than decrease it. Moreover, as Brewin [15] points out, traumatic events do not always involve fear but can be associated with other emotions (e.g. guilt, shame) that lead to differentiated patterns of symptoms. This situation, which frequently occurs among survivors of IPV, may require additional cognitive interventions to the standard treatment with exposure, something that is contemplated in the three programs mentioned.

The challenge, therefore, is to identify the essential elements for treatment while modifying the intervention to make it possible for women to participate and reduce the significant dropout rates (reducing the length, dose or frequency; incorporating additional specific elements…). Similarly, there is a clear need to identify the objectives and priorities of women and adapt treatment to them, including determining what makes it possible for women to be involved in treatment over time in a safe way and developing culturally specific approaches [3]. In addition, the question arises as to whether these treatments are aimed at the trauma recovery or the reduction of trauma symptoms (in fact, most studies measure the outcome by evaluating the effectiveness in reducing the symptoms of PTSD and depression). In this regard, several studies have shown the relationship between PTSD symptomatology and the risk of reabuse in IPV through symptoms of emotional dullness [16, 17]. From this point of view, adequate intervention on PTSD symptoms (particularly on dullness symptoms) would be imperative for the prevention of future reabuse [12].

In recent years, our research has been focused on the analysis of the characteristics of the narrative (and, therefore, of the memory) of women victims of intimate partner violence about the traumatic event they have experienced. The frame of reference for this approach is the cognitive model of trauma memory. The aim is to further develop efficient and effective intervention strategies adapted to the specific problems and needs of this type of victim. When analysing the relationships between the characteristics of the traumatic narratives and the evolution of the victims, it was found that the way in which they reported the trauma allowed the prediction of the development of different types of psychological symptoms [18, 19]. In summary, the results suggest that people who tend to re-experience the emotions linked to the traumatic event during the narrative show a greater immersion in the memory and present more severe symptoms of PTSD. Unfortunately, it is difficult to draw conclusions about the specific nature of the relationship between the way memory is represented and PTSD. It is unclear whether the emotional intensity linked to a memory is the cause of the development of symptoms or if it is the people with the worst evolution who re-experience their emotions more intensely. However, from the first formulations about the functioning of memory, there is the conviction that memory is a primarily constructive process where interpretation plays a crucial role. This implies assuming that memories are created and shaped through subsequent revaluations [20], so it is highly likely that traumatic memories and the emotional response to them are mutually reinforcing. Thus, individual differences in the ability to experience negative emotions with high intensity may be partly responsible for greater accessibility of traumatic memories. Rubin et al. [20] state that this accessibility can lead to frequent re-evaluations of the memory of the event, which can occupy a central place in the personal history of the individual. The high centrality given to the event will contribute, in turn, to maintaining its memory over time, as well as its emotional impact, thus facilitating continuous reassessments. In this way, centrality, accessibility and posttraumatic symptoms could form a vicious circle. The avoidance symptoms of PTSD will illustrate the unsuccessful efforts of the person to break this relationship and reduce the accessibility of traumatic memories.

The study also revealed that the emotional state of the victim is not only related to traumatic memories but also related to the way in which positive events are remembered. To date, narrative studies of PTSD have focused on analysing the content of traumatic memories, and the characteristics of happy memories have been taken into account mainly for comparative purposes. Fernández-Lansac and Crespo [18, 19] suggest that linguistic aspects of positive narratives (i.e. length and speed of speech) can more accurately predict the trajectory of victims than aspects related to the traumatic narratives themselves; differences have been observed in the characteristics of the memories of positive events between women who were victims of violence and women not exposed to trauma [21].

This finding may imply a change in approach by proposing that, although a good adjustment after the trauma depends largely on how the traumatic event is remembered, the way in which the rest of life events are remembered also plays a determining role. Additionally, the degree to which different types of memories compete in the construction of the personal history and identity of the victim can determine posttrauma outcomes. In this study, the impact of positive experiences on the biography of the participants was not studied, but it could be hypothesized that a greater centrality of these experiences would be associated with a better evolution. In fact, although Bernsten et al. [22] found no relationship between the centrality of positive events and the development of posttraumatic symptoms, other studies [23, 24] have highlighted the influence of the importance with which both negative and positive memories are perceived in posttraumatic growth. Moreover, Bernard et al. [23] found that the centrality given to positive events was associated with measures of adaptive functioning, such that the recovery of these memories could serve as a coping mechanism in difficult situations and probably help prevent the effects of stress.

Similarly, Contractor et al. [25] presented a proposal for approaching PTSD that advocates the incorporation of techniques that focus on positive autobiographical memories into trauma-focused treatments. This model was recently updated [26] and was tested in a pilot study [27], and the results showed that the narration of specific positive memories had beneficial impacts on the severity of PTSD symptoms. From this proposal, techniques focused on positive memories would have an effect on the reduction in the severity of PTSD in three ways: (1) direct effects on the severity of PTSD (e.g. through the increase in positive affect and the decrease in negative affect, the development of adaptive cognitions and the increase in the specificity of autobiographical memory), (2) increased effects of trauma-focused interventions (e.g. decreased fear of discussing memories related to trauma and increased preparedness to begin a trauma-focused intervention), and (3) effects on the ease and effectiveness of the processing of traumatic memories (i.e. through an increase in the availability of information and positive experiences that can help to modify the emotional structures related to the trauma, also replacing positive memory to trauma memory as the main point of reference and positively influencing identity and self-concept). Additionally, the incorporation of positive memories has a potential effect in reducing dropout rates (which is important in trauma-focused treatments) and improving treatment adherence and therapeutic alliance.

However, the effect of focusing on positive memories remains to be explored, and it is necessary to continue developing and evaluating intervention proposals focused on the processing of specific positive memories, which are conceived as complementary to trauma-focused interventions. Moreover, this type of proposal is an interesting alternative to solving some of the problems detected in the available interventions for the treatment of trauma in female survivors of IPV.

### Objectives

The *general objective* of the study is to develop and evaluate the efficacy and effectiveness of an evidence-based psychological intervention program focused on trauma that incorporates positive memories into treatment programs for women who have survived IPV. This general objective is divided into the following *specific objectives*:

1. Design of an empirically validated psychological intervention program for women who have survived IPV and have posttraumatic symptoms that incorporates the approach of positive memories into a cognitive-behavioural treatment program focused on trauma.
2. Application and evaluation of the effectiveness of the program focused on positive memories in women who have survived IPV and have posttraumatic symptoms, considering the following:
  Posttraumatic symptoms, including general posttraumatic symptoms and PTSD symptoms
  Variables associated with violence, including experience of violence (physical, sexual and psychological), acceptability of violence; victim blaming attitudes; and motivation for leaving the relationship
  Meanings associated with trauma, including thoughts about the self, the world and the future; self-concept and identity; and centrality of trauma
  Positive and negative affect and regulation of positive and negative affect
  Associated symptoms, including anxiety, depression, self-esteem, emotional dysregulation, and general health

To determine the efficacy, the following will be assessed:

- The effects achieved by the program focused on positive memories compared to a control group on the waiting list
- The differential effects of the cognitive-behavioural treatment program with and without the incorporation of the module corresponding to the approach of positive memories
- The effects achieved by the program focused on positive memories over time (considering follow-up periods of up to 1 year)
- The clinical significance of the changes obtained by the treatment

(3) Evaluation of the implementation and effectiveness of the program, assessed through:
  Adherence to treatment (estimated by the rates of rejections, dropouts and fulfilment of tasks)
  The satisfaction of women with treatment
  Therapists’ satisfaction with the treatment

The *complementary objective* of the project is the development and, where appropriate, translation and validation of instruments for the evaluation of the different variables considered in the study for which there are no versions and adaptations for the Spanish population.

## Methods/design

We used the SPIRIT reporting guidelines [28].

### Design

The design is a single-blind, randomized controlled trial (RCT) comparing two trauma-focused cognitive-behavioural treatment (with and without positive memories’ module) for female survivors of IPV and a wait-list condition (superiority trial). A multigroup design (3 groups) will be used with repeated measures with five levels (pretreatment, posttreatment and follow-ups of 3, 6 and 12 months) for the experimental groups and two levels (pre and post) for the control group. Consequently, the three treatment groups are as follows: (a) waiting list control group (WL), (b) cognitive-behavioural treatment (CBT), and (c) cognitive-behavioural treatment with a focus on positive memories (CBT-M +).

Assessors of treatment outcomes will be blinded to the experimental conditions. To avoid unplanned imbalances in the size of the three groups and a loss of statistical power, a block randomization procedure will be used.

### Setting

Participants will be recruited in community settings, specifically through several women's and IPV support organizations, institutions, and services around Madrid (Spain). Women who attend these centres for attention because of IPV will be referred by professionals and assessed to establish the eligibility criteria.

### Eligibility criteria

We adopt the WHO definition of IPV as ‘any behaviour within an intimate relationship that causes physical, psychological or sexual harm to those in the relationship’ [29]. Participants are eligible if they (1) are women; (2) are over 18 years old; (3) are proficient in the use of the Spanish language; (4) are or have been victims of IPV; (5) have experienced IPV at least one month before the start of the trial; (6) present symptoms of PTSD according to DSM-5 criteria as determined by the Global Assessment of Posttraumatic Stress Scale 5 (EGEP-5; in Spanish: Evaluación Global de Estrés Postraumático 5; Crespo et al. [30]); and (7) have voluntarily agreed to take part in the study.

Participants will be excluded if they (1) show suicide risk as assessed by item 9 (scores > 1) of the Spanish version of the Beck Depression Inventory II—BDI-II [31]; (2) present conditions that could prevent compliance with the treatment (namely, abuse of alcohol or drugs, cognitive impairment or illiteracy in Spanish); (3) have been diagnosed or suffer from psychotic disorders; and (4) have received trauma-focused treatment within the past 6 months.

### Sample size

To determine the target sample size, we considered the effect sizes found in the literature (specifically, the overall effect size—omnibus—calculated in the meta-analysis by Arroyo et al. [5]), which ranges from 0.71 to 1.33. Considering an alpha of 0.05 and a statistical power level of 0.80, a minimum group size of 31 was established. Taking a conservative estimate of the expected dropout rate of 30%, a sample size of 45 women per group will be the target, indicating a total of 135 participants across the 3 experimental conditions (CBT, CBT-M + and WL control group).

### Interventions

The CBT-M + to be developed and evaluated in this project involves the incorporation of the focus on positive memories to the CBT focused on trauma for women victims of IPV previously developed by Labrador et al. [13, 14, 32]. In the experimental groups of this study, both treatments will be applied, that is, the original CBT and the CBT-M +. The two treatments will be applied in group format (6–8 women in each group) in 8 weekly sessions of 80–100 min in duration. Both treatments are detailed below, and the session-by-session application is summarized in Table 1.

**Table 1:** Session-by-session summary of the CBT and CBT-M+

Session (minutes)	CBT	CBT-M +
1 (100 min)	• Presentation and establishment of work standards • Presentation of the intervention and therapeutic objectives • Psychoeducation on abuse and intimate partner violence and its consequences • Signing of the therapeutic contract • Identification of individual objectives • Breathing control training	• Presentation and establishment of work standards • Presentation of the intervention and therapeutic objectives • Psychoeducation on abuse and intimate partner violence and its consequences • Signing of the therapeutic contract • Identification of individual objectives • Breathing control training
2 (95 min)	• Task review • Training to improve self-esteem and cognitive reevaluation • Increase in rewarding activities • Breathing control training	• Task review • Training to improve self-esteem and cognitive reevaluation • Increase in rewarding activities • Breathing control training
3 (90 min)	• Task review • Training to improve self-esteem • Introduction to the exposure technique • Breathing control training	• Task review • Training to improve self-esteem • Introduction to the exposure technique • Breathing control training
4 (80 min)	• Task review • Introduction to the exposure of the history of abuse • **Introduction to the exposure of neutral images** • Warm-up: visualization exercise • Breathing control training	• Task review • Introduction to the exposure of the history of abuse • **Introduction to the exposure of positive memories** • Warm-up: visualization exercise • Breathing control training
5 (95 min)	• Task review • Exposure to history of abuse • Processing of exposure to history of abuse • Breathing control training • **Exposure to the neutral image** • **Processing of exposure to the neutral image**	• Task review • Exposure to history of abuse • Processing of exposure to history of abuse • Breathing control training • **Exposure to positive memory** • **Processing of exposure to positive memory**
6 (95 min)	• Task review • Exposure to history of abuse • Processing of exposure to history of abuse • Breathing control training • **Exposure to the neutral image** • **Processing of exposure to the neutral image**	• Task review • Exposure to history of abuse • Processing of exposure to history of abuse • Breathing control training • **Exposure to positive memory** • **Processing of exposure to positive memory**
7 (100 min)	• Task review • Exposure to history of abuse (hot spots) • Processing of exposure to history of abuse • Breathing control training • **Exposure to the neutral image** • **Processing of exposure to the neutral image**	• Task review • Exposure to history of abuse (hot spots) • Processing of exposure to history of abuse • Breathing control training • **Exposure to positive memory** • **Processing of exposure to positive memory**
8 (90 min)	• Task review • Review of therapeutic objectives • Relapse prevention • Farewell and follow-up planning	• Task review • Review of therapeutic objectives • Relapse prevention • Farewell and follow-up planning

In bold are the differential aspects between both treatments

#### CBT

As mentioned, the CBT to be used is based on the intervention protocol for women who have survived IPV with posttraumatic symptoms developed by the Labrador group and whose efficacy for the reduction of posttraumatic and associated symptoms has already been evaluated across various studies that have examined the following aspects: the effect of the order of the components (cognitive re-evaluation and exposure) [33], the effect of the inclusion or exclusion of the exposure technique [34], individual application vs. group [35, 36] and the application to immigrant women [37].

Of the different versions that have been tested of the reference CBT, in this study, the version that includes the exposure technique will be used (compared to the version that replaces it with training in communication skills) and that uses the order Cognitive Re-evaluation + Exposure (versus the Exposure + Cognitive Re-evaluation option). In addition, given the objective of the study, an extended application of the exposure technique was included (to integrate it with the exposure to the memories of other types of events) following the guidelines of Foa et al. [38].

Consequently, the CBT applied here includes the following modules: (a) psychoeducation, providing the participants information about IPV and its emotional consequences; (b) exercises to control arousal by diaphragmatic breathing; (c) activity scheduling to improve mood; (d) specific techniques to improve self-esteem and re-evaluating biased cognitions; (e) exposure techniques in imagination focused on the memory of IPV situations (including hot-spot exposure); and (f) relapse prevention. In addition, to ensure equivalence between the two programs, the evocation of neutral images in the same spaces and times dedicated in the CBT-M + to the evocation of positive memories is introduced in the general scheme of the CBT.

The first session includes presentations and the establishment of the treatment rules in addition to some information about the violence and about the intervention format. The other sessions start with a brief ‘check in’ and review of the homework, followed by some new elements (cognitive-behavioural skills) to discuss and practice. The specific content for each session is as follows: (1) psychoeducation (session 1), (2) exercises of diaphragmatic breathing (sessions 1–7), (3) activity scheduling (session 2), (4) techniques to improve self-esteem and cognitive re-evaluation (sessions 2–3), (5) exposure techniques (sessions 3–7) and (6) neutral memory evocation (sessions 4–7). Finally, session 8 is focused on relapse prevention. At the end of the first seven sessions, women receive written material that outlined the topics from the session, as well as exercises to be done as homework until the next session. Furthermore, evaluation instruments are applied to assess the satisfaction with the session and the state of the participants (OASIS and ODSIS). Additionally, once the session is over, the therapist completes the instruments for assessing her own satisfaction with the session and the adjustment to the treatment manual during it.

#### CBT- M+

This treatment incorporates a module focused on positive memories into the CBT. In its design, it was taken into account that, since the module would be integrated into the CBT, it would have to be brief, with a development between 1–3 sessions, in order not to prolong the program (something that could go in detriment of adherence). After a detailed review of positive memory intervention techniques [39], the broad-minded affective coping (BMAC) intervention proposed by Tarrier [40] was taken as a reference. This intervention focuses on experiencing positive memories through mental imagery exercises and on experiencing the positive emotions associated with these memories. In its application, participants retrieve an event associated with enjoyment, fun, and happiness. Thereafter, participants are taken through guided imagery of positive memories (engaging all the senses) and re-experiencing of the associated emotions. The original technique consists of one to three sessions, each lasting between 20 and 30 min. A 3-session face-to-face version of the technique was developed and initially tested in a sample of 31 female university students [41].

Although, as pointed by Contractor et al. [25], there is no evidence yet on whether it would be more pertinent to apply positive memories at the beginning and then move on to focus on the trauma, from a clinical point of view, and always taking as a reference the CBT in which the memory module is integrated, it was considered convenient to apply it in combination with the exposure technique, paying particular attention to the fact that positive memories do not become a resource for the escape/avoidance of the exposure itself. To correct this problem, the evocation of positive memories is proposed in an integrated manner with exposure to traumatic memories. Consequently, the application of the module of positive memories within the program is done in the same sessions as the exposure (i.e. 4–7). Moreover, it is proposed that the application in each session of the positive memories after the exposure could lead to a more positive affect after the sessions, in addition to favouring the control of their use as an escape strategy from the exposure. Regarding the content, positive memories not related to trauma are chosen to facilitate therapeutic work and be susceptible to obtaining a greater immediate effect.

For the application of both programs, two manuals have been developed, which in accordance with the guidelines of Carroll and Nuro [42] include the following: general description and reasoning of the treatment, conceptualization of the problem addressed, treatment objectives, specification and definition of the interventions to be included, specification of the content of the sessions, and general treatment format.

The therapist in charge of delivering the intervention is in possession of the certification currently required by legal standards for this task in Spain (i.e. official post-Grad clinical training, Master in General Health Psychology).

Prior to the application of the treatments in the clinical trial and the drafting of the final version of the treatment manuals, a pilot study was conducted in which both treatment modalities were applied in two groups of women between April and June 2021.

### Measures

**Fig. 1:**
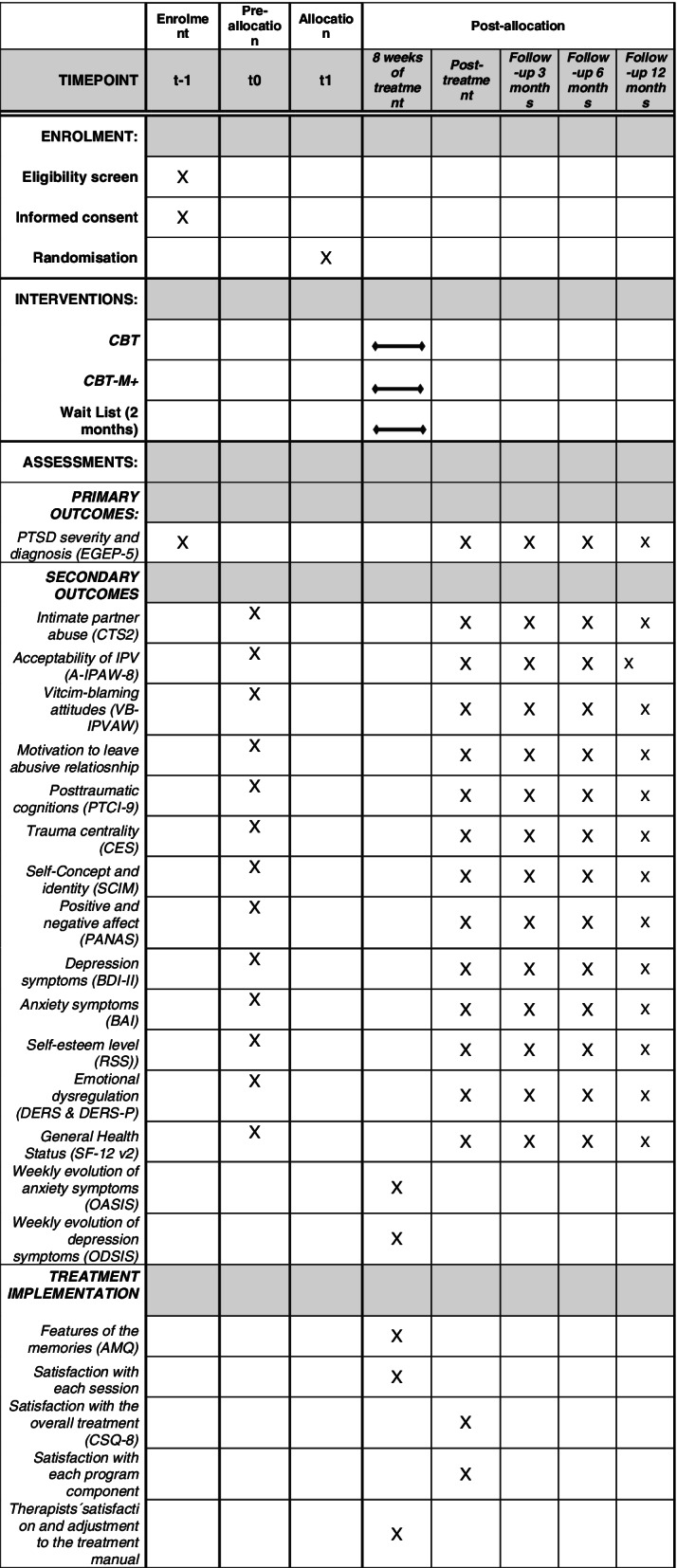
SPIRIT figure of MEMPOSITIV study protocol

The instruments include an initial characterization interview and primary and secondary outcome measures; moreover, treatment implementation variables are also considered. The overview of the assessment is shown in Fig. 1.

#### Characterization interview

In the pretreatment assessment, an interview is conducted to collect data on sociodemographic characteristics (e.g. age, work situation, level of education), the history and characteristics of the abuse and its consequences (e.g. complaint, restraining order…), social and psychological support and substance use. In the posttreatment assessment and in the follow-ups, an abbreviated version of the interview is conducted to assess the changes that have occurred in each of these areas since the last evaluation.

#### Primary outcomes

##### PTSD severity and diagnosis

The primary outcome is the change in the DSM-5 PTSD symptoms. These symptoms will be assessed with the Global Assessment of Posttraumatic Stress Scale 5 (EGEP-5; in Spanish: Evaluación Global de Estrés Postraumático - 5; Crespo et al. [30]), a self-reported measure that assesses the overall severity of PTSD symptoms and provides scores for the DSM-5 clusters for PTSD as well as PTSD diagnosis. The EGEP-5 shows high internal consistency for all severity symptom scales (ranging from 0.72 to 0.86) and for the total (0.91).

#### Secondary outcomes

##### Physical, psychological and sexual intimate partner abuse

Physical, psychological and sexual intimate partner abuse is measured using the Spanish version of the Conflict Tactics Scales version 2 (CTS2) [43] developed by Loinaz et al. [44]. It consists of 78 items that measure the degree to which the members of a couple are involved in physical or psychological attacks on each other, as well as the use of reasoning and negotiation to resolve conflicts. In the present study, only the part in which it is indicated that the partner has suffered each of these episodes will be used (39 items). The psychometric properties of both the original scale and the Spanish version are satisfactory.

##### Acceptability of intimate partner violence

Acceptability of intimate partner violence is measured using the Acceptability of Intimate Partner Violence Against Women short form scale (A-IPVAW-8, Martín-Fernández et al. [45]). The A-IPAW-8 is composed of 8 items in which respondents rate the acceptability of a set of men's behaviours towards their female partners. It has adequate internal consistency and a stable one-factor latent structure.

##### Victim-blaming attitudes

Victim-blaming attitudes is measured using Victim-blaming Attitudes in Cases of Intimate Partner Violence against Women Scale Short Form (VB-IPVAW, Martín-Fernández et al. [46]). This instrument is composed of five items assessing the tendency to blame female victims of IPV. It has been shown to have satisfactory psychometric properties.

##### Motivation to leave the abusive relationship

Motivation to leave the abusive relationship is measured using an ad hoc generated questionnaire (MLAR) with 9 yes/no questions about the possibility of leaving the relationship in progressively graver situations (from ‘in case of questioning about the time you spend with family and friends’ to ‘in case of danger or harm to your children’). Psychometric validation of this questionnaire is ongoing.

##### Posttraumatic cognitions

Posttraumatic cognitions are measured using the Brief Version of the Posttraumatic Cognitions Inventory (PTCI-9; Wells et al. [47]) with the Spanish version elaborated ad hoc taking the items of the translation by Blanco et al. [48]. The PTCI-9 is composed of 9 items across three subscales: Negative Cognitions About the Self, Negative Cognitions About the World and Self-Blame. The PTCI-9 total and subscale scores showed strong internal consistencies.

##### Trauma centrality

Trauma centrality is measured using the Centrality of Event Scale (CES; Bernsten and Rubin [49]), which measures the extent to which a traumatic memory or event forms a central component of an individual’s personal identity and a reference point for the attribution of meaning to other experiences. The reduced 7-item version will be used. Psychometric validation of the Spanish version of this scale is ongoing. The English version has been shown to have satisfactory psychometric properties.

##### Self-concept and identity

Self-concept and identity are measured using the Self-Concept and Identity Measure (SCIM; Kaufman et al. [50]). The SCIM is a 27-item, self-report measure developed to assess identity consolidation and clinically relevant identity disturbance. It has three subscales: identity consolidation, identity disturbance and lack of identity. The English version has been shown to have satisfactory psychometric properties. Psychometric validation of the Spanish version of this tool is ongoing.

##### Positive and negative affect

Positive and negative affect are measured using the Spanish version of the Positive and Negative Affect Schedule (PANAS; Watson, et al. [51]) developed by Sandín et al. [52]. The PANAS consists of two scales designed to measure PA and NA. Respondents are asked to read 20 words describing a series of feelings and emotions and indicate the extent to which they tend to feel them on a Likert scale. The psychometric properties of both scales of the PANAS are satisfactory.

##### Depressive symptoms

 Depressive symptoms are assessed with the Spanish version of the Beck Depression Inventory-II (BDI-II) [31] developed by Sanz et al. [53]. The BDI-II is the most widely used tool to measure depressive symptoms. It consists of 21 items that assess the global level of depression and the changes over time. The score ranges from 0–63 (higher scores indicate more severe symptoms). The Spanish version has been shown to have satisfactory psychometric properties.

##### Anxiety symptoms

Anxiety symptoms are assessed with the Spanish version of the Beck Anxiety Inventory (BAI) [54] developed by Sanz and Navarro [55]. The BAI is commonly used to measure the presence and severity of anxiety symptoms. It consists of 21 items; the score range is 0–63 (higher scores showing more severe symptoms). The internal consistency of the Spanish version is satisfactory.

##### Self-esteem level

Self-esteem level is measured using the Spanish version of Rosenberg’s Self-Esteem Scale (RSS) [56] developed by Echeburúa and Corral [57]. It assesses women’s levels of self-esteem, which is a person’s feelings of self-satisfaction and self-acceptance. The Spanish version has been shown to have satisfactory psychometric properties.

##### Emotional dysregulation

Self-esteem level is measured by the Spanish version of the Difficulties in Emotion Regulation Scale (DERS; Gratz and Roemer [58]) developed by Hervás and Jódar [59]. This 36-item self-report measure with a 5-point Likert scale is used to assess individuals' typical levels of emotion dysregulation across six domains, and it also includes an overall score. The DERS has been shown to have good psychometric properties [58, 60].

The Difficulties in Emotion Regulation Scale – Positive (DERS-P; Weiss et al. [61]) is a 13-item self-report measure with a 5-point Likert scale that is used to measure clinically relevant difficulties in regulating positive emotions. The DERS-P was modelled after the original DERS [58] and comprises three specific domains as well as an overall score. It has demonstrated good psychometric properties [61]. Psychometric validation of the Spanish version of this scale is ongoing.

##### General health status

 General health is measured using the Spanish version of the 12 Item Short Form Health Survey Version 2 (SF-12 v2; Ware et al. [62]) developed by Alonso [63]. The SF-12 is one of the most widely used instruments to assess health-related quality of life grouped into two major dimensions: physical health and mental health. SF-12v2, in its original version, has shown internal consistency values of 0.88 for the physical health component and 0.82 for the mental health component [64].

##### Weekly evolution of anxious symptoms

 Weekly evolution of anxiety symptoms is measured using the Spanish version of the Overall Anxiety Severity and Impairment Scale (OASIS; Norman et al. [65]) developed by Osma et al. [66], at the end of each of the 8 sessions. It consists of five items and evaluates the frequency, intensity and interference of anxious symptomatology in the person's life over the last week. The Spanish version has been shown to have good internal consistency.

##### Weekly evolution of depressive symptoms

Weekly evolution of depressive symptoms is measured using the Spanish version of the Overall Depression Severity and Impairment Scale (ODSIS; Bentley et al. [67]) developed by Osma et al. [66], at the end of each of the 8 sessions. It consists of five items and evaluates the frequency, intensity and interference of sadness/depression in the person's life over the last week. The Spanish version has been shown to have good internal consistency.

#### Treatment implementation

##### Features of the memories

The Autobiographical Memory Questionnaire (AMQ) [68] is used to determine the features of the memories (about maltreatment and about positive events) that are evoked in the exposure and the positive memories module, respectively. It consists of several questions concerning the processes involved in remembering an event. For this study, two versions were generated: one with 9 questions that collect information about the particular memory that has just been evoked, specifically, the way the person coped with the event and the integration and coherence of this memory in life history and personal identity, and another version with 12 items that adds three questions that characterize the event in general (when and how many times it happened and what it is like). AMQ has proven useful for measuring basic processes used in autobiographical memory construction and its association with posttraumatic adaptation [69]. Psychometric validation of the Spanish version of this tool is ongoing.

##### Satisfaction with each session

Satisfaction with each session is measured using an ad hoc generated questionnaire based on Marín et al. [70] at the end of each of the 8 sessions. It consists of four items that assess the following using a visual analogue 10-point scale: satisfaction with session; perceived usefulness of the session; mood and emotional state; and group cohesion.

##### Satisfaction with the overall treatment

Satisfaction with the overall treatment is measured using the Spanish version of the Client Satisfaction Questionnaire (CSQ-8; Larsen et al. [71] developed by Echeburúa and Corral [57]. This questionnaire assesses women’s satisfaction with the program and the treatment. The CSQ-8 has been shown to have high internal consistency and concurrent validity in mental health outpatient settings [72].

##### Participants’ satisfaction with each program component and aspect

Participants’ satisfaction with each program component and aspect is measured using an ad hoc generated questionnaire completed anonymously at the end of the last session. It consists of several visual analogue 10-point scales that assess satisfaction with each component of the program (i.e. breathing, self-esteem, pleasure activities, exposure to maltreatment, exposure to hot spots, exposure to positive/neutral memory, and homework) and with several issues related to its implementation (i.e. therapist, co-therapist, assessor, initial information session, reminder of the sessions, collective coffee, group environment, facilities, information provided by the centre and materials).

Additionally, in the final session, the participants complete a qualitative and anonymous evaluation of the different components and aspects of the program.

##### Therapists’ satisfaction and adjustment to the treatment manual

Participants’ satisfaction with each program component and aspect is measured using an ad hoc generated questionnaire complemented by the therapist at the end of each of the 8 sessions. It consists of six items with Likert scales ranging from 0-10 that assess satisfaction with the session, contents, participation and assimilation of contents, and adjustment to the protocol and the timing. Moreover, information about the real duration of the session and about possible incidences is recorded.

To provide a guide through which the therapist can verify the adherence of the session to the treatment protocol, a checklist is available for both intervention modalities. In this list, the 8 treatment sessions are outlined, with the different components of each session, the essential contents of each of them and the materials, questionnaires and records necessary for the correct application of the session.

### Procedure

Once the participants are recruited through the collaborating centres, the members of the research team contact them, and an appointment is made to carry out the initial screening. Those participants who meet the inclusion criteria are informed of the project and the possibility of participating in it. If they agree to participate, informed consent will be signed, and an appointment will be made with them to carry out the initial evaluation. Those women who do not meet the inclusion criteria or who show their desire not to participate in the project are referred to the centres so that they can receive assistance.

The evaluation is carried out by trained psychologists who are blinded to the treatment modality. Once the initial evaluation is completed, the participants are assigned to a therapeutic group based on their time availability. The determination of the treatment modality of each group is determined randomly using the random number generator of the University of Granada (https://www.ugr.es/~jsalinas/Aleatorios.htm).

The application of the treatment is preceded by an informative-motivational session in which the participants are informed of the characteristics of the program and the presentation of the research team is made.

The treatment is developed in 8 weekly sessions, and thus, it lasts approximately 2 months. In the case of the participants assigned to the waiting list control group, a new evaluation is performed (with the same instruments as in the pretreatment evaluation) after 2 months (i.e. period equivalent to the duration of the treatments), and they are offered the possibility of participating in the treatment in case of acceptance of such participation. The therapists are also psychologists with clinical training who do not participate in the initial evaluation of the participants or in the subsequent evaluations and who are trained in the application of the different treatment modalities. In each treatment group, there are a therapist and a co-therapist in charge of assisting the therapist in carrying out the tasks during the session and in the delivery and collection of materials. The co-therapists are students with training in clinical psychology.

Once the treatment is finished, a new evaluation is carried out to be performed in the first 14 days after the last treatment session following the same guidelines as in the pretreatment evaluation and including in addition to the same battery applied in the pretreatment (except for the question identification), the questionnaire of satisfaction with the treatment. Likewise, follow-ups at 3, 6 and 12 months are performed.

Both the evaluators and the therapists and co-therapists are women to facilitate the therapeutic alliance.

All evaluation and treatment sessions are carried out in the reference centres of each participant, except in those cases in which they express their desire to do so in the Faculty of Psychology of the Complutense University of Madrid (something that is always offered as a possibility).

For the supervision of the therapy sessions to increase adherence to the protocols, one session is audio recorded in each intervention group (which is specified in the informed consent form). The determination of the session to be recorded in each intervention group is randomly assigned by a third person through the application of the online randomization program (https://www.augeweb.com/azar/).

Supervision is performed by an experienced senior clinical psychologist, who has been involved in the development of the currently tested program and the prior pilot study, as well as in the ongoing decision-making process regarding refinements to the protocol. He receives weekly information provided by the psychologist delivering the program, give guidance regarding adherence to the protocol and provide troubleshooting as needed. Communication between the supervisor and the delivering psychologist is done mostly through e-mails and, to a lesser extent, online supervision sessions, but more immediate exchanges through the telephone also occur when needed.

Treatment fidelity is assessed through the revision of the randomly recorded sessions (one for each group), with a focus on proper adherence to the times allotted for each treatment component in the session. When program delivery is been found to stray from the delivery-as-intended, proper feedback is provided, and follow-up contacts regarding this guidance occur. The development of a quantitative measure for treatment fidelity is currently underway.

Based on the literature, no harmful effects are expected from the application of the applied psychological treatments and procedures. However, if adverse reactions or changes that may pose a danger to the participants are identified, their participation in the study will be terminated and, where appropriate, they will be offered the possibility of receiving psychological care through the reference entities or the Complutense University of Madrid.

After treatment and assessment completion, an external researcher that has not participated in the assessment and therapy implementation carries out the data entry and coding of the information, being blind to participants’ identity. Both paper based and electronic data entry are used. After performing range check for data values, statistical analyses will be addressed by an expert in methodology, who remains blind to the therapeutic process.

Results will be communicated to the public, participants, healthcare professionals and other relevant groups via publications, databases, social media and sponsor and research group website.

### Statistical analysis

For the graphic description of the flow of the participants throughout the protocol, the CONSORT diagram is used. The intention-to-treat (ITT) method will also be used. In the treatment of the missing values, it will be checked if the loss of data occurs completely at random using Little’s MCAR test, and then, the estimation of the missing values will follow the maximum likelihood method through the EM algorithm [73].

As a step prior to determining the effectiveness of the intervention programs, the homogeneity of the groups will be analysed, taking into account all the variables evaluated and using *χ*^2^ tests and ANOVAs according to the type of variables.

To determine if the treatment is better than the control group, one-factor (type of intervention) ANOVAs and ANCOVAs will be performed for each of the scores in the posttreatment (or second pre measure for the control group on the waiting list) of the continuous treatment outcome variables, using in the ANCOVAs the value of the pretreatment evaluation as covariance. In the case of ANOVAs, the DSM or Games-Howel tests will be used for post hoc analyses depending on whether the assumption of equality of variances is met. In the case of significant differences in the ANCOVAs, the groups will be analysed in pairs using new ANCOVAs. For those variables that do not adjust to normality, the corresponding nonparametric tests will be applied (i.e. Kruskal–Wallis and Mann–Whitney *U* for pairwise comparisons). For the qualitative variables, *χ*^2^tests will be used.

To determine the changes over time, the two basic moments will be analysed first (i.e. the changes from pretreatment to posttreatment or the second premeasure for the control group). Repeated-measures ANOVAs will be performed in a mixed design with an intergroup factor with three levels (the two treatments plus the control group) and an intrasubject factor (pre and post measures). In those variables that do not adjust to normality, the corresponding nonparametric tests will be applied (i.e. Friedman). Additionally, in this case, for the qualitative variables, *χ*^2^ tests will be used.

In a second moment of the analysis of the changes over time, the changes between the pre, post and follow-ups will be analysed only for the two experimental groups. For this, an ANOVA will be applied for each of the outcome variables, including an intergroup factor (with two levels: CBT and CBT-M +) and an intrasubject or repeated measures factor, with five levels (i.e. pre, post, 3 months, 6 months and 12 months). In the application of these ANOVAs, the assumption of sphericity will be verified using the Mauchly test, and where appropriate, the Huynh-Feldt correction will be used. Likewise, ANCOVAs of one factor (type of treatment) will be performed for each outcome variable, using the value of the pretreatment evaluation as covariance.

To determine the differences between the two treatments, the intergroup differences and the interactions between time and type of treatment obtained in the different ANOVAs and ANCOVAs mentioned in the previous point will be addressed.

In all analyses, the Holm–Bonferroni correction will be used to adjust the level of significance for multiple comparisons.

To establish the clinical significance of the changes obtained, *χ*^2^ tests will be used considering the data relative to those variables that present a cut-off point capable of discriminating the clinical improvement of the participants. Likewise, the relative risk of presenting problems of one experimental group with respect to the other at different times of measurement will be calculated. In addition, the McNemar related proportions will be calculated to verify if the percentage of problems is the same or different in the pretreatment and in the different subsequent measurement moments, and the Jacobson-Truax method will be applied by calculating the index of reliable change. This analysis will be supplemented by the calculation of the effect size of each variable.

In the specific analysis of the reabuse or revictimization variables, it is expected that the scores show high asymmetry and kurtosis, so they will be recoded as dichotomous variables (presence vs. absence for each type of violence or abuse) in accordance with the procedure described by Johnson et al. [12].

To establish the effectiveness of the intervention program, adherence to treatment will be evaluated considering the percentage of dropouts in each group, the session in which dropouts occur, the number of sessions attended by the women who complete the treatment and the fulfilment of homework. For this, the two treatment groups will be compared in these variables through *χ*^2^ tests or, where appropriate, one-way ANOVA.

Finally, for the analysis of the variables related to therapeutic success, multiple regression (for the prediction of the values of quantitative variables) or logistic regression (for the prediction of therapeutic success vs. failure) will be applied. In addition, to the extent that each variable allows, moderation and mediation analyses will be performed with the PROCESS macro for SPSS using the procedure described by Hayes [74].

## Discussion

This article described the study protocol of an RCT comparing a trauma-focused CBT for female survivors of IPV with versus without the incorporation of positive memory evocation. Consequently, this study first analyses the effect of adding a positive memory module to a trauma-focused CBT for female survivors of IPV.

The primary aim of the study is to try to correct some of the difficulties detected in the treatment of this type of population, improving adherence to treatment, decreasing dropout rates and achieving significant improvements in both the symptomatology and the well-being of the women treated in comparison with other therapeutic options focused exclusively on trauma. In this way, the therapeutic proposal presented here is framed in the model of complex trauma by Courtois [75], particularly enhancing the recovery of trauma with the development of a more integrated and emotionally modulated autobiographical narrative and a gradual reorientation to the present and future, with the creation of new meanings and purposes, and with the reconstruction of a life that is no longer defined by trauma and its effects. Consequently, the treatments will be compared on a broad range of outcomes in addition to PTSD symptoms, including meanings associated with trauma, affect, associated symptoms, quality of life, attitudes towards violence and revictimization.

The present study will contribute to the advancement in the development of intervention programs increasingly adapted to the characteristics and needs of people with complex interpersonal traumas that involve a change and a vital break in the personal future. In addition, it presents the value of starting from a novel approach in which traditional models and treatments focused on trauma are complemented with interventions focused on positive memories. In this way, the results of this project can become a fundamental point of reference to determine the potential and real scope of this new proposal, which, although it has gained relevance in recent years [39], is still in an initial phase. In short, this study introduces a broader view of trauma and its consequences and searches to enhance women’s recovery.

In the design and implementation of the study, the guidelines outlined in the CONSORT statement (http://www.consort-statement.org), as well as the specific guidelines for trauma-focused interventions proposed by the International Society of Traumatic Stress Studies—ISTSS [38] were followed. In addition, this study has several strengths. First, there was a pilot study prior to the clinical trial. In relation to the experience of the pilot study, several modifications were introduced in the study protocol that are key to its evolution, including the introduction of reminder calls of the session to each participant of the group the day before the session, the introduction of a space for informal exchange between the participants of each group at the end of each session, the introduction of a motivational-informative talk at the beginning of the program, the modification of exercises and instructions for some of the contents included in the treatment manual and the incorporation of the co-therapist to assist the therapist in the management and streamlining of group tasks.

Second, the application and evaluation of the positive memory module as a step prior to its incorporation into the CBT [41] is also a strength. This study allowed us to establish the effect of the module while providing information on the guidelines to be followed in its application.

Third, the trial not only focuses on changes in PTSD but also focuses on other symptoms that are common and significant among survivors of IPV (e.g. depression, anxiety, self-concept, emotional dysregulation). In this sense, it is worth mentioning the inclusion of measures to establish the effect of the program on attitudes towards IPV and, above all, on the probability of revictimization, as well as the possible mediating effect of these variables in the reduction of posttraumatic symptomatology, which is especially relevant for the recovery of these women. In addition, the incorporation of this large number of variables, especially those related to the processes, will allow for the establishment of the effect achieved by the treatment as well as the underlying mechanisms and predictors of treatment outcome, which, conversely, will provide insight into how the treatment works and for whom.

Fourth, the study incorporates various instruments to control and evaluate that the applied techniques achieve the desired effect. Thus, in the application of the modules of positive memories and neutral images, the assessment of the emotional state caused by the evocation (i.e. degree of discomfort and degree of well-being) is incorporated, as well as the level of activation that it arouses. This evaluation allows us to verify whether the evocation of neutral images is truly neutral (i.e. low discomfort, low well-being and medium activation) and whether the evocation of positive memories is truly positive (e.g. low discomfort, high well-being and medium or high activation).

A final strength of the study is its application in community settings, which allows, on the one hand, an increase in the ecological validity and representativeness of the results with respect to the usual work with survivors of IPV and, on the other hand, the promotion of the dissemination of the program among professionals who work in direct care of this population, which is one of the basic motivations of this project.

The main difficulty of the study will be the adherence of the participants to the intervention programs. In fact, this is one of the problems that is at the base of the approach of this study and one of the fundamental reasons for the development of the intervention program proposed here. Although the proposed program tries to obviate this problem, adapting as much as possible to the specific circumstances of women, it cannot be forgotten that in most cases, the victims of violence who request assistance (whether legal or psychological) are frequently immersed in a period of vital changes, which can make it difficult for them to attend the sessions and perform the various tasks at home that may be entrusted to them.

Regarding the possible difficulties inherent to the characteristics and application of the program itself, the possible use of positive memories as an avoidance strategy is proposed, as mentioned above. On the other hand, the selection of positive memories not related to the trauma can sometimes be problematic in women who have been immersed in a violent relationship for many years and for whom many of the most positive experiences of their life (e.g. the birth of a son or his wedding day) are related in one way or another to the aggressor.

Regarding the limitations, it should be noted that, as it is proposed, this program is accessible only to women without functional illiteracy in Spanish and with a certain sociocultural level, which limits to some extent its possibilities of application (e.g. immigrant women with little command of Spanish, who, according to the data, are a population at high risk). It would be convenient, therefore, once the effectiveness of the program is established, to make adaptations that allow its application in this type of women.

## Trial status

Recruitment started on 15 September 2021 and is still ongoing. The estimated completion date is March 2023. Protocol version 10 March 2022.

## Data Availability

The datasets analysed during the current study and statistical code are available from the corresponding author on reasonable request, as is the full protocol.

## Abbreviations

A-IPVAW: Acceptability of Intimate Partner Violence Against Women
AMQ: Autobiographical Memory Questionnaire
ANCOVA: Analysis of covariance
ANOVA: Analysis of variance
BAI: Beck Anxiety Inventory
BDI: Beck Depression Inventory
CBT: ; CBT-M +: Cognitive-Behavioural therapy plus positive memories module
CES: Centrality of Events Scale
CONSORT: Consolidated Standards of Reporting Trials
CSQ: Client Satisfaction Questionnaire
CTS: Conflict Tactics Scales
DERS: Difficulties in Emotion Regulation Scale
DERS-P: Difficulties in Emotion Regulation Scale—Positive
EGEP-5: Global Assessment of Posttraumatic Stress Scale
IPV: Intimate partner violence
ITT: Intention to treat
OASIS: Overall Anxiety Severity and Impairment Scale
ODSIS: Overall Depression Severity and Impairment Scale
PANAS: Positive and Negative Affect Schedule
PTCI: Posttraumatic Cognitions Inventory
PTSD: Posttraumatic stress disorder
RCT: Randomized controlled trial
RSS: Rosenberg Self-esteem scale
SCIM: Self-Concept and Identity Measure
SF-12 v2: Health Survey—Short Form version 2
VB-IPVAW: Victim-blaming Attitudes in Cases of Intimate Partner Violence against Women
WHO: World Health Organization
